# Insights into 6‐Methylsalicylic Acid Bio‐assembly by Using Chemical Probes

**DOI:** 10.1002/ange.201509038

**Published:** 2016-02-02

**Authors:** James S. Parascandolo, Judith Havemann, Helen K. Potter, Fanglu Huang, Elena Riva, Jack Connolly, Ina Wilkening, Lijiang Song, Peter F. Leadlay, Manuela Tosin

**Affiliations:** ^1^ Department of ChemistryUniversity of WarwickLibrary RoadCoventryCV4 7ALUK; ^2^ Department of ChemistryUniversity of CambridgeLensfield RoadCambridgeCB2 1EWUK; ^3^ Department of BiochemistryUniversity of Cambridge80 Tennis Court RoadCambridgeCB2 1GAUK; ^4^ School of BiosciencesThe University of BirminghamBirminghamB15 2TTUK

**Keywords:** Chemische Sonden, Dehydratase, Iterative Polyketidkatalyse

## Abstract

Chemical probes capable of reacting with KS (ketosynthase)‐bound biosynthetic intermediates were utilized for the investigation of the model type I iterative polyketide synthase 6‐methylsalicylic acid synthase (6‐MSAS) in vivo and in vitro. From the fermentation of fungal and bacterial 6‐MSAS hosts in the presence of chain termination probes, a full range of biosynthetic intermediates was isolated and characterized for the first time. Meanwhile, in vitro studies of recombinant 6‐MSA synthases with both nonhydrolyzable and hydrolyzable substrate mimics have provided additional insights into substrate recognition, providing the basis for further exploration of the enzyme catalytic activities.

## Graphical abstract

**Chemische Sonden**, die mit Ketosynthase(KS)‐gebundenen Biosynthesezwischenstufen reagieren, wurden genutzt, um die iterative Polyketidsynthase 6‐Methylsalicylsäure‐Synthase (6‐MSAS) in vivo und in vitro zu untersuchen. Aus der Fermentierung pilzlicher und bakterieller 6‐MSAS‐Wirte in Gegenwart von Kettenabbruchsonden wurde ein ganzes Spektrum von Biosyntheseintermediaten isoliert (siehe Beispiele) und charakterisiert.

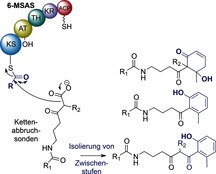

Together with peptidic molecules, polyketide natural products constitute one of the most abundant sources of bio‐derived and bio‐inspired pharmaceuticals: these include widely utilized antibiotic and antitumor agents (for example, erythromycin and doxorubicin) and cholesterol‐lowering (statin) drugs.^1^ Polyketide biosynthesis resembles that of fatty acids in the use of decarboxylative Claisen condensation to generate enzyme‐bound β‐keto thioester intermediates, which are variably processed and ultimately converted to highly diversified products in structure and function. Polyketide synthases (PKSs) can be distinguished as either modular or iterative: the former (best known as type I modular) are assembly lines comprising multiple sets of domains (modules), with each catalyzing at least one round of chain extension and downstream translocation for further processing. For these PKSs the module order and composition determine the sequence of biosynthetic events,^2^ which can be predicted and manipulated to generate novel compounds.^3^ Conversely iterative synthases are constituted by single enzymes (type I and III) or clusters of discrete proteins (type II) that harbor a limited set of catalytic domains iteratively utilized for intermediate chain growth and elaboration. The rapid and unpredictable nature of substrate processing makes the investigation of iterative synthases much more challenging compared to their modular counterparts. Type I iterative polyketide synthases (iPKSs) most closely resemble fatty acid synthases in their domain organization and modus operandi. They are typical of fungi,^4^ although an increasing number have been reported in bacteria.^5^ Type I iPKSs utilize a diverse range of acyl building blocks (for example, acetate, hexanoate, and benzoate) as starter units for polyketide chain building, and have been classified as non‐reducing (NR), partially reducing (PR), or highly reducing (HR) according to the degree of ketone moiety processing occurring throughout product assembly.^4^ Tailoring modifications can occur during and after assembly to yield the final bioactive molecules.^8^ Established mechanisms of iPKS product release include cyclization and hydrolysis mediated by thioesterase (TE) and Claisen cyclase thioesterase (CLC‐TE) domains, thioester reduction,^4^, ^5^ pyrone formation,^6^ and product transfer to a nonribosomal peptide synthetase (NRPS) assembly line.^7^ Important mechanistic insights on iPKSs have been gathered by the in vitro reconstitution of enzyme activity with putative synthetic substrates,^9^ by genetic manipulation,^10^ and by analysis of protein site‐occupancy using advanced mass spectrometry of PKS‐bound precursors.^11^ However, key details of the timing of biosynthetic transformations and the basis for substrate discrimination for iPKSs remain elusive, and new tools are needed to uncover them.

6‐methylsalicylic acid (6‐MSA, **1**, Scheme 1) was the first polyketide to be biosynthetically investigated.^12^ It is produced in various fungi including *Penicillium patulum*, where it is a precursor to the toxin patulin.^13^ 6‐MSA is also a key structural moiety of promising antibiotic and anticancer agents, such as chlorothricin, maduropeptin, and neocarzinostatin.^14^ The iPKS 6‐methysalicylic acid synthase (6‐MSAS) was first purified from *P. patulum* and characterized as a 188 kDa tetrameric protein.^15^ Early labeling experiments established that 6‐MSAS requires one acetyl‐CoA (**2**) and three malonyl‐CoA molecules (**3**, Figure 1 A) to generate 6‐MSA.^16^ Analysis of its gene cluster revealed it encodes one polypeptide chain harboring ketosynthase (KS), acyltransferase (AT), dehydratase (DH), ketoreductase (KR), and acyl carrier protein (ACP) domains^17^ as in a vertebrate fatty acid synthase. Mechanistic studies of purified 6‐MSAS using substrate/intermediate analogues and enzyme inhibitors,^18^ as well as enzyme mutagenesis,14a, 19 have led to two distinct biosynthetic proposals: in the first, DH‐catalyzed dehydration of a 3‐hydroxytriketide intermediate is followed by a further round of chain extension, *trans* to *cis* isomerization of a double bond, aromatization, and finally thioester hydrolysis (Scheme 1 a).18c, 19a In the second, a 3‐hydroxytriketide intermediate is directly extended to a 5‐hydroxytetraketide, which cyclizes, dehydrates, and aromatizes prior to final product release (Scheme 1 b).18c, 19b A recent study of the 6‐MSAS‐like enzyme ATX from *Aspergillus terreus* has supported this second route and provided evidence of involvement of a so‐called thioester hydrolase (THID) domain in product release.19b The THID domain comprises the previously identified dehydratase (DH) domain together with an adjacent region termed the interdomain (ID) linker, originally identified as a core domain required for subunit–subunit interaction within ATX.19a THID has been shown to catalyze 6‐MSA release from a mutant form of ATX (H972A, which would inactivate the DH function); it also catalyzes hydrolysis of the *N*‐acetylcysteamine thioester of 6‐MSA.19b This suggests that enzyme‐catalyzed dehydration of a 3‐hydroxytriketide intermediate is not necessary for 6‐MSA formation. However, in the absence of direct evidence for the biosynthetic intermediates involved, it remains unclear whether triketide dehydration takes place and whether the sole role of THID is the hydrolytic release of enzyme‐bound 6‐MSA.

**Figure 1:**
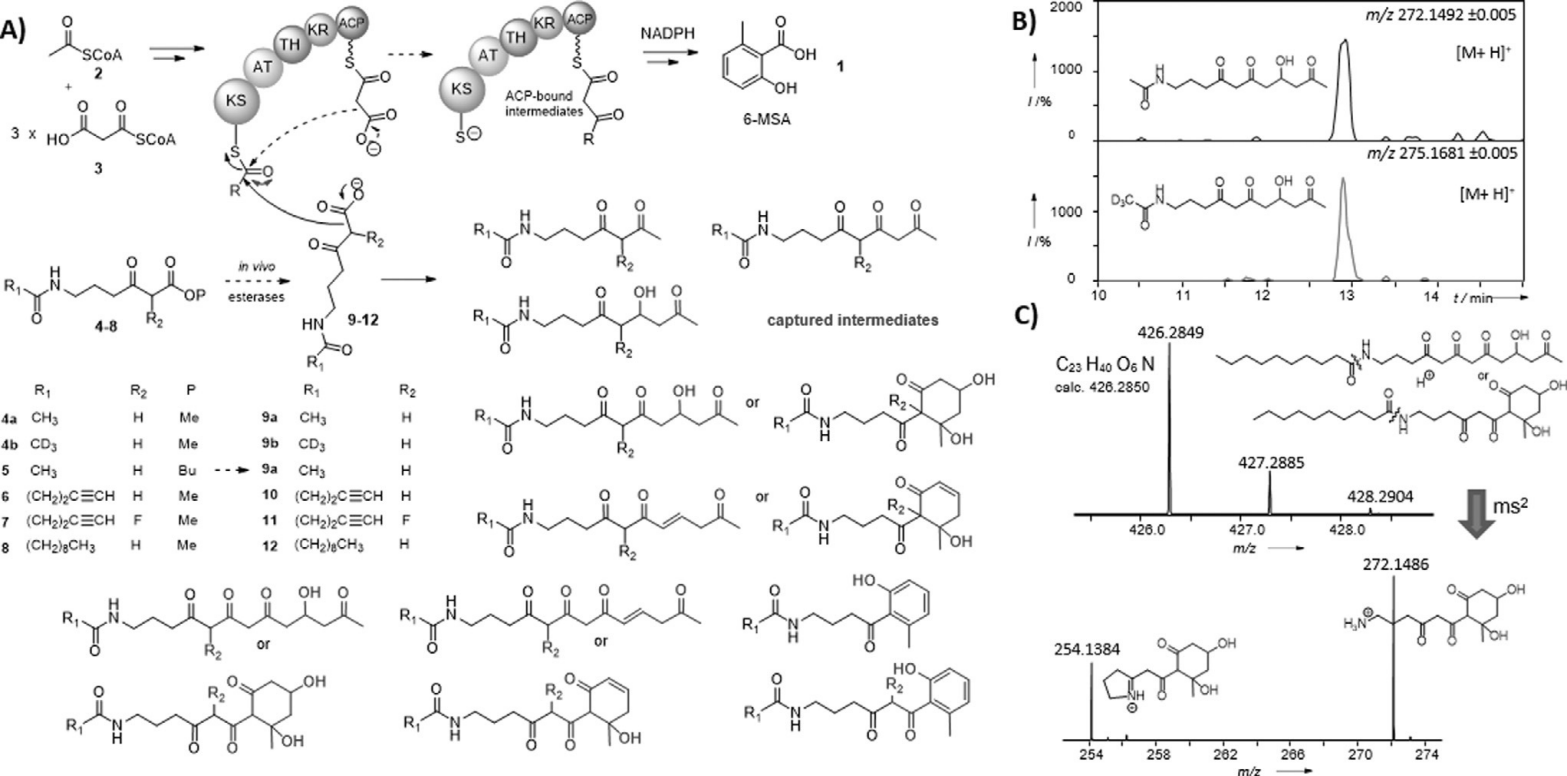
A) Chain termination probes **9**–**12**, generated in vivo from the hydrolysis of the corresponding esters **4**–**8**,^21^ compete with ACP‐bound malonate to off‐load 6‐MSAS‐bound intermediates in *P. patulum*, *E. coli* heterologously expressing 6‐MSAS,^22^ and *S. antibioticus* DSM40725 (Supporting Information, Tables 1S–3S); B) LC‐ HR‐MS detection of putative hydroxytetraketides captured from *P. patulum*; C) HR‐MS^n^ analyses of a putative hydroxypentaketide resulting from the off‐loading of a KS‐bound hydroxytetraketide in *P. patulum*.

**Scheme 1:**
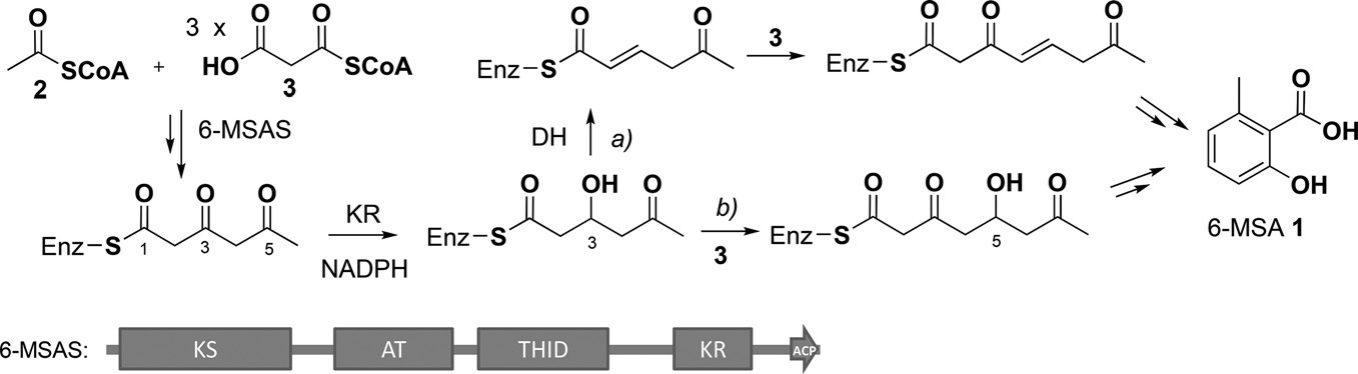
Overview of biosynthetic hypotheses leading to 6‐methylsalicylic acid (6‐MSA, **1**): a) enzymatic dehydration of a 3‐hydroxytriketide followed by further chain extension leads to a dehydrated enzyme‐bound tetraketide, eventually giving **1**; b) formation of a 5‐hydroxytetraketide eventually leading to **1** via TH‐mediated hydrolysis.18c, 19b 6‐MSAS comprises ketosynthase (KS), acyltransferase (AT), thioester hydrolase (THID),19b ketoreductase (KR), and acyl carrier protein (ACP) domains.

To obtain a complete mechanistic picture of 6‐MSA assembly, we have used chain‐termination probes for the capture and identification of polyketide intermediates.^20^, ^21^ By competing with ACP‐bound malonate extension units for the growing polyketide chain, the chemical probes react with enzyme‐bound intermediates and off‐load them for LC‐MS characterization (Figure 1). The use of these tools has already allowed fresh insights into the timing and the mechanism of modular assembly‐line biosynthesis in vitro^20^ and in vivo,^21^ and, more recently, has unveiled novel opportunities for the generation of unnatural polyketide derivatives.21c We initially used our intermediate‐capturing probes for in vivo studies on fungal and bacterial strains harboring 6‐MSAS genes, including the natural 6‐MSA producer *P. patulum*, an engineered *E. coli* host strain heterologously expressing *P. patulum* 6‐MSAS (*E. coli* BAP1 pKOS007‐109),^22^ and *S. antibioticus* DSM40725 (producer of chlorothricin).14a Each strain was grown in the presence of substrates **4**–**8**, which are hydrolyzed in vivo to the corresponding carboxylates **9**–**12** (Figure 1 A; Supporting Information, Figure 2S).^20^

The overall outcome of these in vivo experiments is illustrated in Figure 1 (for details, see the Supporting Information, Tables 1S–3S and following figures). In most of the ethyl acetate extracts from both fungal and bacterial hosts, a series of trapped intermediates, including diketides, triketides, reduced triketides, and a range of putative hydroxy, dehydrated, and aromatized tetraketides, were identified by HR‐LC‐MS: these would directly reflect the nature of ACP‐bound substrates in 6‐MSA assembly. Besides, putative hydroxy, dehydrated, and aromatized pentaketides arising from the off‐loading of 6‐MSAS‐bound tetraketides were also identified (Figure 1 C and the Supporting Information). All the captured intermediates, absent in control samples, were characterized by MS^n^ analysis, showing diagnostic peaks resulting from the loss of *N*‐acyl chains and subsequent cyclic imine formation (Figure 1 C and the Supporting Information). For the majority of the putative tetraketides and pentaketides, multiple peaks were observed: these may arise from isomerization, cyclization and dehydration events which can be spontaneous or enzyme‐catalyzed. A distinction between hydroxy, dehydrated, and aromatized advanced species was made on the basis of variable LC retention times as well as of detected accurate masses. On the same basis, distinct species with masses corresponding to dehydrated triketides could not be identified. From the lack of direct evidence for dehydrated triketides and the identification of the putative hydroxy tetraketides and pentaketides, it appears that, whether the PKS is of fungal or of bacterial origin, route b) of Scheme 1 is followed.

Nonetheless, to seek additional confirmation of these insights and further dissect 6‐MSAS catalytic activities, we also utilized recombinant *P. patulum* 6‐MSAS from heterologous *E. coli* BAP1 host strain,^23^ as well as an additional mutant form of the enzyme (6‐MSAS H958A) bearing an alanine in place of a histidine in the THID active site for in vitro assays.^22^ The capture of biosynthetic intermediates in vitro proved much more challenging than in vivo. Using probes **9 a**–**b** (generated from pig liver esterase‐ assisted hydrolysis of **4 a**–**b**),19b only intermediates from two rounds of chain extension were consistently identified in the ethyl acetate extracts of 6‐MSAS assays (Supporting Information, Figure 43S). When recombinant 6‐MSAS was primed with acetoacetyl‐CoA instead of acetyl‐CoA in the attempt to improve advanced intermediate capture, the accumulation of a possibly dehydrated triketide was observed (Supporting Information, Figure 46S).

To investigate whether this species could have been enzymatically formed, a racemic 3‐hydroxytriketide substrate mimic **13** was synthesized in five steps from **4 a** (Supporting Information) and tested as a substrate for both 6‐MSAS and its H958A mutant. In neither case was **13** enzymatically dehydrated (Figure 2 A; Supporting Information, Figure 47S); rather, we found that purified **13** dehydrated over long‐term storage. Surprisingly, when 6‐MSAS and the H958A mutant were incubated with the *N*‐acetylcysteamine thioester of 6‐MSA **14,** previously utilized to probe the THID function in ATX,^12^ no free 6‐MSA was generated (Figure 2 B; Supporting Information, Figure 48S). An *N*‐decanoyl thioester analogue **15** was synthesized as an additional substrate, with the idea of utilizing a long acyl chain to mimic the phosphopantetheine cofactor of ACP. However, **15** was also resistant to hydrolysis by either enzyme (Supporting Information, Figure 49S). This unexpected outcome suggests that 6‐MSAS differs from ATX in that covalent attachment of tetraketide intermediates to the ACP or coenzyme A might be necessary for their processing. Alternatively, this may indicate that readily aromatized thioesters are not true substrates for THID domains. The results reported herein strongly point towards no enzymatic dehydration taking place at a triketide stage of 6‐MSA assembly, so loss of water must occur at the tetraketide stage. If this is enzyme‐catalyzed, the configuration of the resulting alkene (from an R‐alcohol, as recently established for the 6‐MSA‐like mellein synthase)^24^ would likely be *trans*. On the basis of multiple peaks observed for dehydrated tetraketides (for example, Supporting Information, Figures 24S), it is tempting to speculate that, along with final thioester hydrolysis, the THID might act as a template domain to aid *trans* to *cis* double bond isomerization, and/or cyclization and aromatization; however, this remains undetermined. Amongst iPKSs with significant homology to 6‐MSAS and leading to 6‐MSA related products, the THID domain is highly conserved (Supporting Information, Figure 50S). Its presence in non‐reducing iPKSs such as the orsellinic acid synthase supports its role in product cyclization and release.14a Intriguingly, a THID is not present in MicC, an iPKS responsible for the formation of 6‐pentasalicylic acid in micacocidin biosynthesis, whose assembly allegedly proceeds similarly to that of 6‐MSA.7b, 25 Although bioinformatic analysis usefully pinpoints the similarities of 6‐MSAS to ATX and other iPKSs (Supporting Information, Figure 51S), it cannot be used to infer subtle differences in substrate recognition and processing between these multienzymes. For this the use of chemical probes appears a promising approach towards a fuller understanding and exploitation of iPKS catalysis. Through the use of the chain termination probes **9**–**12** we have indeed gathered the first direct and comprehensive evidence for the course of substrate processing on a type I iterative PKS. The probes were successfully used in a fungal host and both Gram‐positive and Gram‐negative bacteria, with the putative intermediates providing preliminary insights into the kinetics of 6‐MSA assembly. In general, the first two rounds of chain extension/processing appear relatively slow, whereas more distinct differences can be observed in advanced intermediate accumulation in the different 6‐MSAS hosts (Supporting Information, Tables 1S–3S and following analyses): these may depend on probe efficiency and uptake to some extent, as well as on specific kinetic programming of product assembly within a particular host. Significantly 6‐MSAS displayed unexpected substrate flexibility, in that it was able to accept malonate surrogates of different chain lengths and bearing various functionalities (including alkyne and fluorine moieties) at different positions and for every round of chain extension, and also to generate novel pentaketide products. This opens the possibility of utilizing iPKSs for the generation of novel unnatural products employing different extender21c as well as starter units.^26^

**Figure 2:**
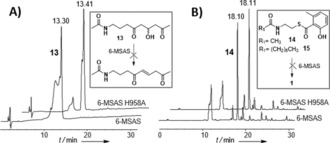
Analytical HPLC analyses showing that: A) the synthetic triketide mimic **13** is not dehydrated by 6‐MSAS nor by its mutant 6‐MSAS H958A and B) the thioester substrates **14** and **15** are not hydrolyzed by 6‐MSAS nor by its mutant 6‐MSAS H958A.

## Supporting information

As a service to our authors and readers, this journal provides supporting information supplied by the authors. Such materials are peer reviewed and may be re‐organized for online delivery, but are not copy‐edited or typeset. Technical support issues arising from supporting information (other than missing files) should be addressed to the authors.

Supplementary
